# Noninvasive differentiation of benign and malignant solid pulmonary nodules using multiparameter dual-layer spectral CT radiomics

**DOI:** 10.1186/s13244-026-02380-8

**Published:** 2026-09-04

**Authors:** Yanyan Wang, Ye Yu, Yicheng Fu, Ying Zhang, Yu Wang, Xiao Yu, Baocong Liu, Yan Zhou, Huawei Wu

**Affiliations:** 1 Department of Radiology, Renji Hospital, School of Medicine, Shanghai Jiao Tong University, 200127 Shanghai, People’s Republic of China; 2 Clinical and Technical Support, Philips Healthcare, 200072 Shanghai, People’s Republic of China; 3 State Key Laboratory of Oncology in South China, Department of Radiology, Sun Yat-sen University Cancer Center, 510060 Guangzhou, Guangdong People’s Republic of China

**Keywords:** Diagnosis, Differential, Lung neoplasms, Radiomics, Tomography, X-ray computed

## Abstract

**Objectives:**

To establish and validate a noninvasive multiparametric radiomics model based on dual-layer spectral CT (DLCT) for distinguishing preoperatively malignant from benign solid pulmonary nodules (SPNs).

**Materials and methods:**

This retrospective study enrolled 441 patients with pathologically confirmed SPNs who underwent preoperative DLCT and were divided into training (*n* = 252), internal test (*n* = 112), and external test (*n* = 77) cohorts. Radiomics features were extracted from conventional and virtual monoenergetic images (40 and 70 keV) and material decomposition images (including iodine density (ID), *Z*-effective atomic number (Zeff), and electron density (ED) maps) in arterial (AP) and venous (VP) phases. Logistic regression was used to construct radiomics models, and a combined clinical-radiomics model was visualized as a nomogram. Subgroup analysis was performed by nodule size (≤ 10 mm vs.  > 10 mm), and diagnostic accuracy was compared with that of two radiologists.

**Results:**

The optimal radiomics model, comprising features from ID in VP and Zeff in both AP and VP, achieved an area under the curve (AUC) of 0.835, 0.804, and 0.772 in the training, internal, and external cohorts, respectively. The combined model (age, lobulation, and ten radiomic features) outperformed the clinical-radiological model in all cohorts (AUC: 0.889 vs. 0.816; 0.865 vs. 0.795; 0.825 vs. 0.743; *p* < 0.05). It maintained strong performance for ≤ 10 mm and > 10 mm nodules, with AUCs of 0.875 and 0.888 (internal test) and 1.000 and 0.794 (external test).

**Conclusion:**

A DLCT-based multiparametric radiomics model, integrated with clinical-radiological features, enables accurate preoperative, noninvasive differentiation between benign and malignant SPNs, including subcentimeter nodules.

**Key Points:**

- **Question:** Accurate preoperative differentiation of solid pulmonary nodules remains clinically challenging because benign and malignant nodules often show overlapping features on conventional CT.
- **Findings:** A combined model integrating multiparameter dual-layer spectral CT radiomics and clinical-radiological features outperformed the clinical-radiological model in differentiating benign and malignant solid pulmonary nodules.
- **Critical relevance statement:** The developed model provides an accurate, noninvasive tool for preoperative differentiation of indeterminate solid pulmonary nodules, supporting clinical management decisions.

**Graphical Abstract:**

**Figure:**
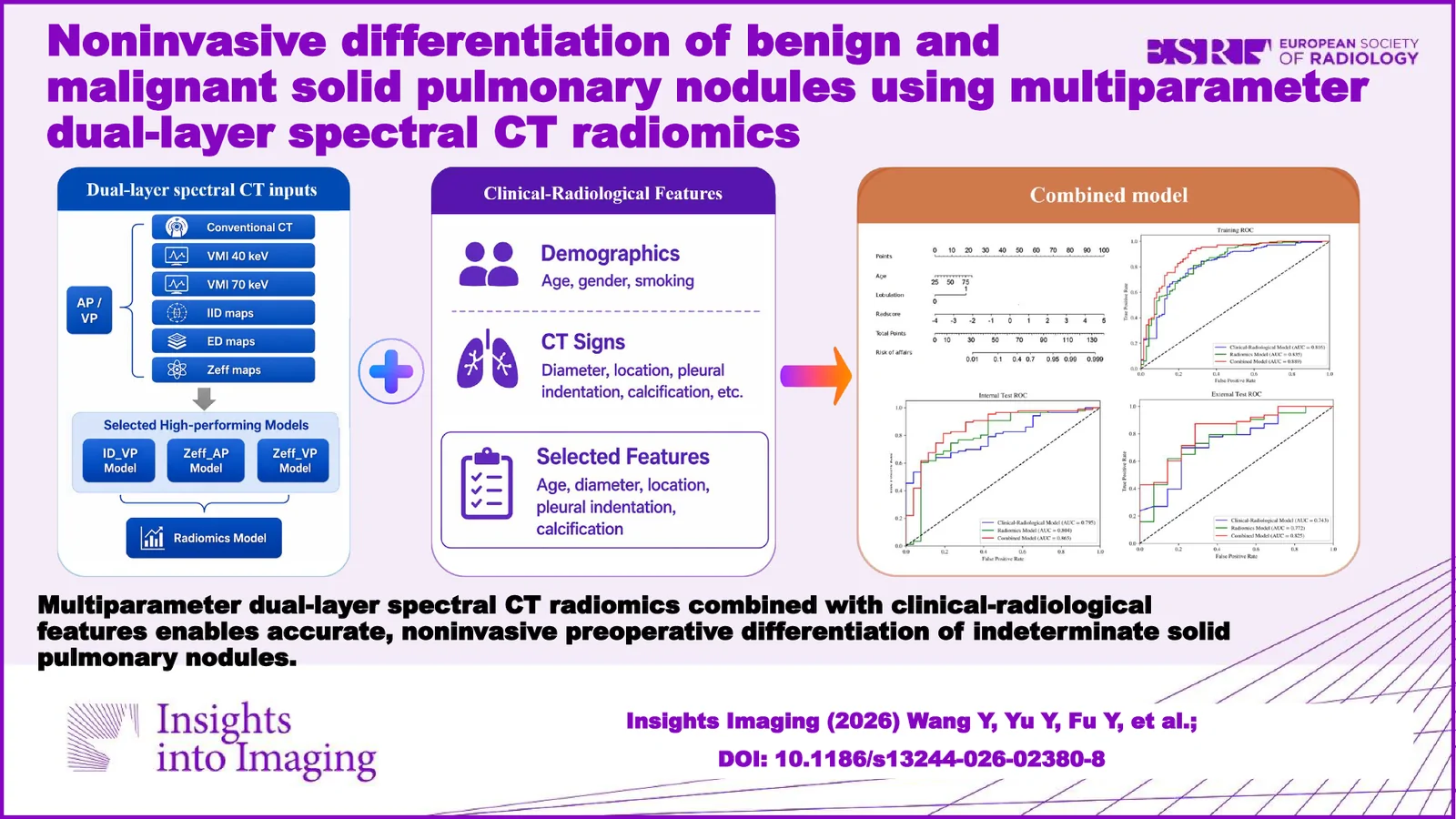

## Introduction

Lung cancer remains the most common cause of cancer-related morbidity and mortality worldwide [1]. As outlined in the 9th edition of the TNM staging system, the 5-year survival rate for patients with stage IA1 non-small cell lung cancer (NSCLC) is 88%, but declines markedly to 21% in stage IIIC cases [2], highlighting the critical importance of early detection. In its early stages, lung cancer often presents as solitary pulmonary nodules [3]. The widespread adoption of high-resolution CT (HRCT) and AI techniques has substantially improved the detection rate of pulmonary nodules [4]. Among density-based subtypes, solid pulmonary nodules (SPNs) warrant particular attention because malignant SPNs exhibit more aggressive biological behavior than subsolid nodules, including faster growth, poorer prognosis, and a higher tendency for early metastasis [3, 5, 6]. However, accurately differentiating benign from malignant SPNs remains a significant clinical challenge.

Conventional CT evaluation, which relies primarily on morphological and enhancement characteristics, often faces challenges due to significant overlap between benign and malignant SPNs, leading to diagnostic uncertainty [7]. Consequently, current guidelines recommend regular follow-up for indeterminate nodules, causing psychological distress and cumulative radiation exposure [8]. Moreover, data from the National Lung Screening Trial indicate that 28% of low-dose CT-detected nodules that were resected proved benign, underscoring the risk of unnecessary surgery [9]. While invasive biopsy and surgery remain diagnostic standards, they carry risks of complications and potential overtreatment [6]. This highlights the pressing need for a noninvasive, reliable biomarker to distinguish benign from malignant SPNs preoperatively.

Dual-layer spectral CT (DLCT) provides additional quantitative material decomposition data beyond conventional CT [10], including spectral curve slope, virtual monoenergetic images (VMIs), iodine density (ID), electron density (ED), and effective atomic number (Zeff). Growing evidence supports the utility of DLCT in various aspects of lung cancer management, including histological subtyping [11], metastasis prediction [12], tumor staging [13], and treatment monitoring [14]. Crucially, the provision of diverse quantitative spectral parameters offers substantial potential to enhance the differentiation of benign and malignant SPNs [15–17]. Considering the constraints of conventional imaging and the growing diagnostic potential of DLCT, there is a compelling need to explore advanced analytic approaches that can fully leverage its multiparametric data.

Radiomics involves the high-throughput extraction of quantitative features from medical images, thereby offering objective characterization of tumor heterogeneity by analyzing subtle patterns that are not discernible to human perception [18, 19]. While conventional CT-based radiomics has been extensively investigated for SPNs differentiation [3, 8, 20, 21], research integrating DLCT with radiomics remains limited. Current DLCT radiomics studies predominantly focus on single-parameter analyses (e.g., 40–70 keV VMIs or ID maps) [7, 22, 23], overlooking the potential synergistic value of integrating multiparametric spectral information. This study aims to develop and validate a machine learning-based radiomics model that integrates multiparametric DLCT data (VMI, ID, ED, Zeff) to preoperatively differentiate benign from malignant SPNs, with histopathologic findings after surgery serving as the reference standard. The model’s performance was also evaluated on sub-centimeter nodules and compared with radiologists' performance to assess its potential as a preoperative, noninvasive decision-support tool for indeterminate SPNs.

## Materials and methods

### Study population

**Fig. 1:**
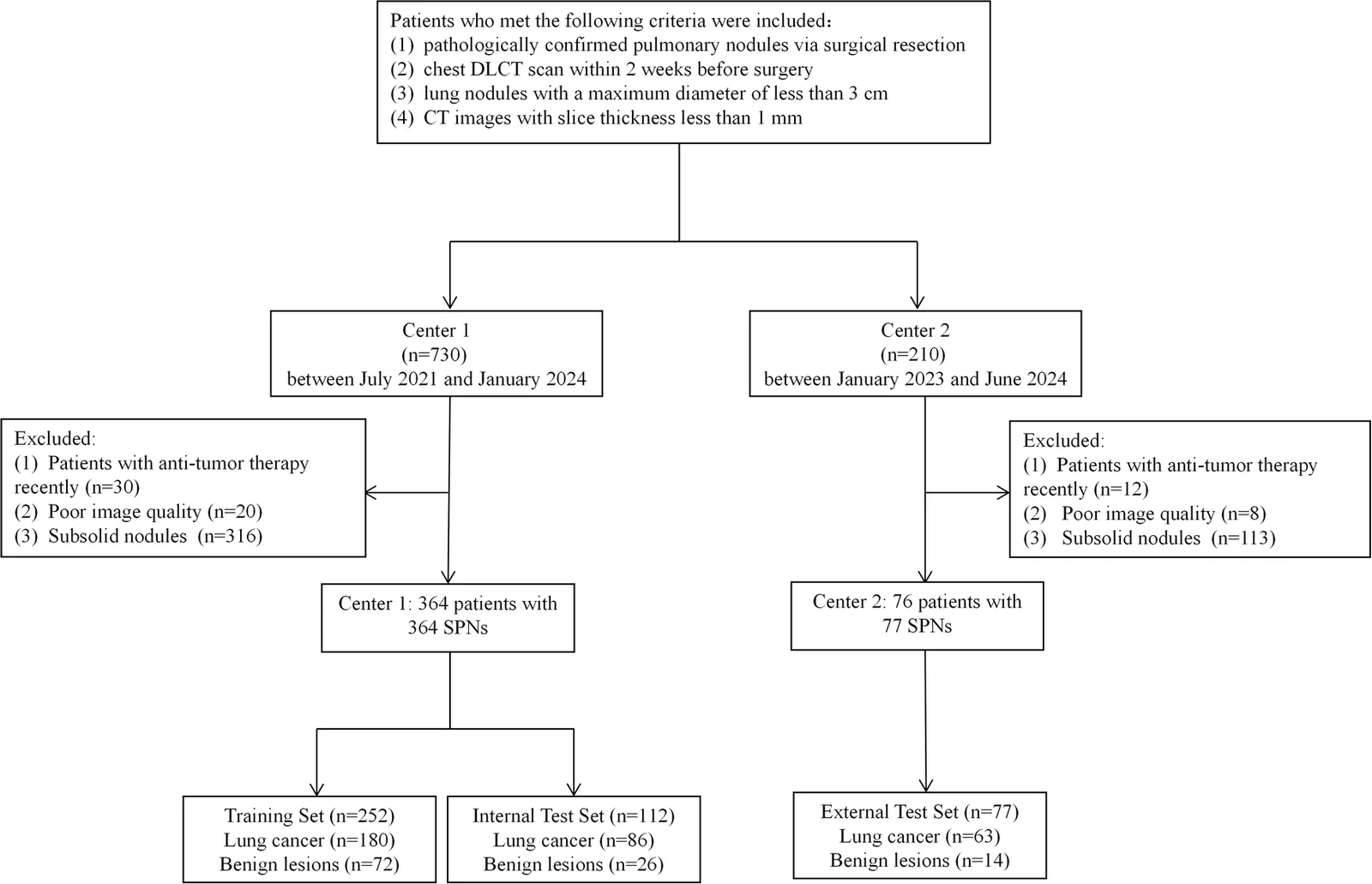
Flowchart of patients’ screening procedure. DLCT, dual-layer spectral CT; SPNs, solid pulmonary nodules

This retrospective study was approved by the institutional review boards of Renji Hospital, affiliated to Shanghai Jiao Tong University School of Medicine (Center 1) (approval number: LY2025-036-B) and Sun Yat-sen University Cancer Center (Center 2) (approval number: B2025-189-01) with waived informed consent. Between July 2021 and January 2024, 364 patients with pathologically confirmed nodules were consecutively recruited from Center 1 and randomly allocated into training and internal test cohorts at a 7:3 ratio. An independent test cohort consisting of 76 patients with 77 nodules was enrolled from Center 2 between January 2023 and June 2024. The patient selection process is summarized in Fig. 1.

Inclusion criteria were: (1) pathologically confirmed pulmonary nodules via surgical resection; (2) preoperative DLCT performed within two weeks before surgery; (3) lung nodules with a maximum diameter ≤ 3 cm; (4) CT images with slice thickness less than 1 mm. Exclusion criteria included: (1) Recent anti-tumor therapy; (2) Severe respiratory or metal artifacts compromising image quality; (3) Subsolid nodules (pure or part-solid).

### CT protocol

All patients underwent unenhanced and dual-phase contrast-enhanced chest scans using a DLCT scanner (IQon Spectral CT, Philips Healthcare). The detailed information on the scanning protocols for Center 1 and Center 2 can be found in S1.

Twelve spectral image sets were reconstructed by a post-processing workstation (IntelliSpace Portal 12, Philips Healthcare) for each patient, comprising conventional images, ID maps, Zeff maps, ED maps and VMI at 40 keV and 70 keV from both arterial and venous phases (VPs).

### Clinical and radiological feature analysis

Clinical data and pathological diagnoses, including age, sex, and smoking history, were collected from electronic medical records. A junior radiologist (Radiologist A, 3 years of experience) initially assessed the CT images, which were subsequently reviewed by an intermediate radiologist (Radiologist B, 8 years of experience). Discrepancies were adjudicated by a senior radiologist (Radiologist C, 25 years of experience). All radiologists were blinded to clinical and pathological information. Images were evaluated using the lung and mediastinal windows. The recorded CT signs were as follows: (1) diameter (the longest dimension measured on axial images in the CT lung window); (2) Nodule location; (3) shape; (4) clear boundary; (5) Additional imaging features: lobulation, spiculation, spinous process, vacuole, cavitation, air bronchogram, vascular convergence, pleural indentation, and calcification [3, 4].

### Radiomics workflow

Image segmentation: CT images from both arterial phase (AP) and venous phase (VP) were imported into ITK-SNAP software (Version 3.80, http://www.itksnap.org). Using the lung window, radiologist A performed semi-automatic segmentation of regions of interest (ROI) based on a thresholding algorithm, followed by manual slice-by-slice adjustments to exclude large vessels, bronchi, chest wall, and mediastinal structures. For reproducibility assessment, 30 randomly selected cases in the training cohort were re-segmented by radiologist A and radiologist B 1 month later. Reproducibility was assessed using intraclass correlation coefficients (ICCs) based on a two-way mixed-effects model (intra-observer) and a two-way random-effects model (inter-observer), both with absolute agreement and single measurement [24]. Only features with both ICCs > 0.75 were retained.

Feature extraction: ROIs delineated on conventional CT images were automatically co-registered to the reconstructed spectral images. The 12 sets of spectral images were preprocessed through isotropic resampling to 1 × 1 × 1 mm³ voxel size, gray-level discretization with a fixed bin width of 25. A total of 1118 radiomic features were extracted per ROI using the PyRadiomics package (version 3.1.0; pyradiomics community), following guidelines from the Image Biomarker Standardization Initiative (IBSI). Before feature selection, *z*-score normalization was applied to standardize all nodule features.

Feature selection: First, features with ICCs ≥ 0.75 were retained to ensure reproducibility. Second, the minimum redundancy maximum relevance (mRMR) algorithm was applied to identify the most informative features, while those with high collinearity (Spearman correlation ≥ 0.8) were excluded. Finally, the least absolute shrinkage and selection operator (LASSO) with five-fold cross-validation was used to select features with nonzero coefficients.

**Fig. 2:**
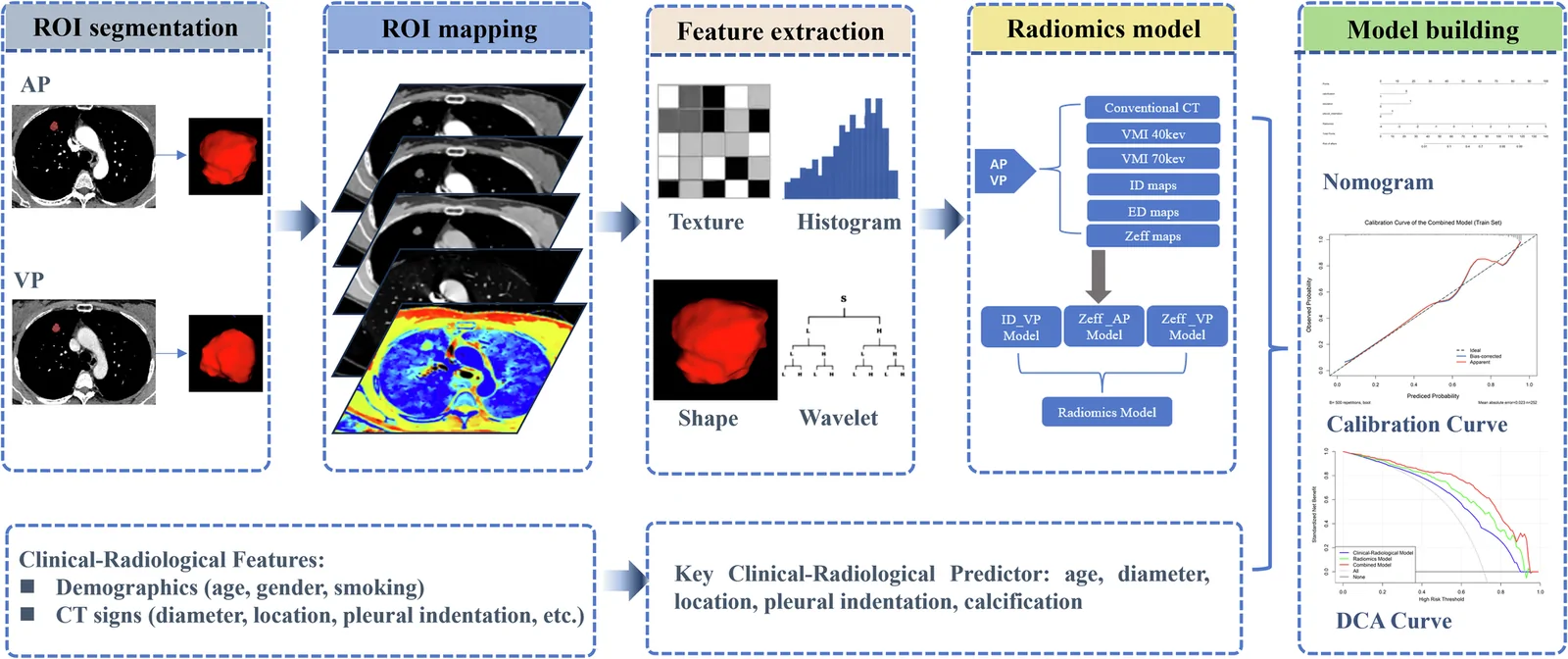
The flowchart of the combined model establishment. AP, arterial phase; VP, venous phase; VMI, virtual monoenergetic images; ID, iodine density; ED, electron density; Zeff, effective atomic number; ROI, regions of interest; DCA, decision curve analysis

Model construction: Twelve single-spectral radiomics models were constructed, each corresponding to one spectral image type from AP and VP. Logistic regression with five-fold cross-validation was used to develop the model in the training cohort. Models with an average area under the curve (AUC) > 0.75 were considered high-performing. To determine the most informative spectral image type, we assessed how often each image type contributed to high-performing models during cross-validation. The top-ranking image types were then selected and combined to develop the multiparametric radiomics model, from which a corresponding Radscore was calculated. A final combined model integrating selected clinical, radiological, and multiparametric Radscore was then built via logistic regression and visualized as a nomogram. The workflow is shown in Fig. 2.

### Diagnostic performance evaluation

Two radiologists (Radiologist B and Radiologist C) independently assessed SPNs in the internal test set. Receiver operating characteristic (ROC) analysis was performed to compare the diagnostic performance of the combined model with that of the radiologists.

### Statistical analysis

Statistical analyses were conducted using SPSS (version 26.0) and R software (version 4.4.2; https://www.r-project.org). The Kolmogorov–Smirnov test was applied to assess the normality of continuous variables. Normally distributed data were presented as mean ± standard deviation, while non-normally distributed variables were expressed as median (interquartile range). Categorical variables were summarized as frequencies and percentages. Levene’s test was used to assess homogeneity of variance. Group comparisons for continuous variables were performed using the independent-samples *t*-test or Mann–Whitney *U*-test, as appropriate, and the chi-square test was used for categorical variables. Univariable and multivariable logistic regression analyses were performed to identify independent predictors among clinical and CT variables. Model performance was assessed using ROC curves, with AUC, accuracy, sensitivity, and specificity reported. The DeLong test was employed to compare AUCs between models. The calibration of the nomogram was evaluated using calibration curves and the Hosmer–Lemeshow goodness-of-fit test. Clinical utility was assessed via decision curve analysis (DCA). Subgroup analysis was performed to evaluate the combined model performance in nodules stratified by size (≤ 10 mm vs. > 10 mm). A *p*-value < 0.05 was considered statistically significant.

## Results

### Patient characteristics

This study enrolled 441 SPNs, comprising 252 nodules in the training cohort (72 benign, 180 malignant), 112 nodules in the internal test cohort (26 benign, 86 malignant), and 77 nodules in the external test cohort (14 benign, 63 malignant). The histological types from Center1 and Center2 are presented in Table S1. Clinical and radiological characteristics are summarized in Table 1. Age demonstrated significant differences in benign and malignant differentiation in the training cohort (*p* < 0.001), while no statistical differences were found in sex and smoking history. In the training cohort, malignant nodules were more likely to exhibit larger diameter, irregular shape, blurred boundary, lobulation, spiculation, vacuole sign, vascular convergence, pleural indentation, and less frequent calcification (all *p* < 0.05).

**Table 1:** Demographic information and CT characteristics of training and internal test, external test sets

Characteristics	Training set (*n* = 252)			Internal testing set (*n* = 112)			External testing set (*n* = 77)		
	Benign (*n* = 72)	Malignant (*n* = 180)	*p*	Benign (*n *= 26)	Malignant (*n* = 86)	*p*	Benign (*n* = 14)	Malignant (*n* = 63)	*p*
Gender			0.054			0.244			0.577
Female	38 (52.8%)	71 (39.4%)		13 (50.0%)	32 (37.2%)		6 (42.9%)	22 (34.9%)	
Male	34 (47.2%)	109 (60.6%)		13 (50.0%)	54 (62.8%)		8 (57.1%)	41 (65.1%)	
Age (years)	54.0 ± 13.7	62.9 ± 11.2	< 0.001^a^	56.6 ± 12.1	63.2 ± 10.2	0.007^a^	58.9 ± 12.1	59.9 ± 9.7	0.758
Smoking history			0.579			0.163			0.276
Yes	16 (22.2%)	46 (25.6%)		4 (15.4%)	25 (29.1%)		4 (28.6%)	28 (44.4%)	
No	56 (77.8%)	134 (74.4%)		22 (84.6%)	61 (70.9%)		10 (71.4%)	35 (55.6%)	
Diameter(mm)	13 (10,17.8)	17(13,23.8)	< 0.001^a^	17 (15,19)	20 (15.26)	0.041^a^	18 (12.3,21.3)	20 (15,26)	0.281
Location			0.092			0.255			0.089
RUL	12 (16.7%)	49 (27.2%)		8 (30.8%)	22 (25.6%)		7 (50.0%)	10 (15.9%)	
RML	6 (8.3%)	21 (11.7%)		2 (7.7%)	7 (8.1%)		1 (7.1%)	6 (9.5%)	
RLL	20 (27.8%)	27 (15.0%)		8 (30.8%)	14 (16.3%)		2 (14.3%)	16 (25.4%)	
LUL	13 (18.1%)	38 (21.1%)		7 (26.9%)	27 (31.4%)		3 (21.4%)	18 (28.6%)	
LLL	21 (29.2%)	45 (25.0%)		1 (3.8%)	16 (18.6%)		1 (7.1%)	13 (20.6%)	
Shape			0.004^a^			0.141			0.265
Round or oval	48 (66.7%)	84 (46.7%)		19 (73.1%)	49 (57.0%)		8 (57.1%)	48 (76.2%)	
Irregular	24 (33.3%)	96 (53.3%)		7 (26.9%)	37 (43.0%)		6 (42.9%)	15 (23.8%)	
Clear boundary			0.002^a^			0.005^a^			0.547
Clear	27 (37.5%)	106 (58.9%)		7 (26.9%)	50 (58.1%)		8 (57.1%)	44 (69.8%)	
Blurred	45 (62.5%)	74 (41.1%)		19 (73.1%)	36 (41.9%)		6 (42.9%)	19 (30.2%)	
Lobulation	39 (54.2%)	158 (87.8%)	< 0.001^a^	12 (46.2%)	78 (90.7%)	< 0.001^a^	7 (50.0%)	53 (84.1%)	0.015^a^
Spiculation	22 (30.6%)	97 (53.9%)	0.001^a^	4 (15.4%)	49 (57.0%)	< 0.001^a^	0 (0.0%)	13 (20.6%)	0.142
Spinous process	9 12.5%)	31 (17.2%)	0.354	5 (19.2%)	19 (22.1%)	0.755	4 (28.6%)	20 (31.7%)	1.0
Vacuole	2 (2.8%)	23 (12.8%)	0.016^a^	1 (3.8%)	8 (9.3%)	0.628	0 (0.0%)	4 (6.3%)	1.0
Cavitation	2 (2.8%)	10 (5.6%)	0.543	2 (7.7%)	7 (8.1%)	1.0	1 (7.1%)	5 (7.9%)	1.0
Air bronchogram	10 (13.9%)	30 (16.7%)	0.586	0 (0.0%)	17 (19.8%)	0.032^a^	1 (7.1%)	7 (11.1%)	1.0
Vascular convergence	9 (12.5%)	44 (24.4%)	0.036^a^	1 (3.8%)	15 (17.4%)	0.157	0 (0.0%)	16 (25.4%)	0.079
Pleural indentation	22 (30.6%)	109 (60.6%)	< 0.001^a^	6 (23.1%)	53 (61.6%)	0.001^a^	4 (28.6%)	45 (71.4%)	0.003^a^
Calcification	9 (12.5%)	9 (5.0%)	0.037^a^	9 (34.6%)	0 (0.0%)	< 0.001^a^	1 (7.1%)	0 (0.0%)	0.182

Data are expressed as numbers (%), or mean ± standard deviation, or medians (interquartile ranges)

*RUL* right upper lobe, *RML* right middle lobe, *RLL* right lower lobe, *LUL* left upper lobe, *LLL* left lower lobe

^a^ Statistically significant

### Clinical-radiological model

Multivariable logistic regression identified age, diameter, lobulation, pleural indentation, and calcification as independent predictors in the differentiation between benign and malignant SPNs (Table 2). The clinical-radiological model achieved AUCs of 0.816 (95% confidence interval (CI): 0.755–0.877), 0.795 (95% CI: 0.711–0.880), and 0.743 (95% CI: 0.594–0.892) in the training, internal validation, and external validation cohorts, respectively (Table 3).

**Table 2:** Univariate and multivariate logistic regression analysis of clinical-radiological characteristics in training sets

Characteristics	Univariate analysis		Multivariate analysis	
	OR (95%CI)	*p*	OR (95%CI)	*p*
Age (years)	1.06 (1.04–1.08)	< 0.001^a^	1.05 (1.02–1.08)	< 0.001^a^
Diameter (mm)	1.12 (1.07–1.18)	< 0.001^a^	1.11 (1.04–1.19)	0.002^a^
Shape	2.29 (1.29–4.05)	0.005^a^	1.14 (0.52–2.52)	0.740
Clear boundary	0.42 (0.24–0.74)	0.02^a^	0.85 (0.39–1.84)	0.673
Lobulation	6.08 (3.19–11.56)	< 0.001^a^	6.65 (2.99–14.79)	< 0.001^a^
Spiculation	2.66 (1.49–4.75)	0.001^a^	1.20 (0.55–2.59)	0.649
Vacuole	5.13 (1.18–22.35)	0.03^a^	5.38 (0.80–36.30)	0.084
Vascular convergence	2.27 (1.04–4.92)	0.039^a^	1.23 (0.47–3.24)	0.678
Pleural indentation	3.49 (1.95–6.26)	< 0.001^a^	3.30 (1.58–6.91)	0.002^a^
Calcification	0.37 (0.14–0.97)	0.043^a^	0.19 (0.05–0.76)	0.019^a^

*OR* odds ratio, *CI* confidence interval

^a^ Statistically significant

**Table 3:** Performance of the clinical-radiologic, radiomics, and combined models in the training, internal test, and external test sets

Model	AUC (95%CI)	Accuracy	Sensitivity	Specificity
Training set				
Clinical-radiologic model	0.816 (0.755–0.877)	0.774	0.783	0.750
Radiomics model	0.835 (0.776–0.893)	0.786	0.811	0.722
Combined model	0.889(0.840–0.938)	0.873	0.928	0.736
Internal test set				
Clinical-Radiologic model	0.795 (0.711–0.880)	0.732	0.791	0.538
Radiomics model	0.804 (0.694–0.913)	0.759	0.802	0.615
Combined model	0.865 (0.782–0.945)	0.804	0.802	0.808
External test set				
Clinical-Radiologic model	0.743 (0.594–0.892)	0.740	0.762	0.643
Radiomics model	0.772 (0.633–0.912)	0.740	0.762	0.643
Combined model	0.825 (0.708–0.942)	0.818	0.841	0.714

*AUC* area under the curve, *CI* confidence interval

### Radiomics model

**Fig. 3:**
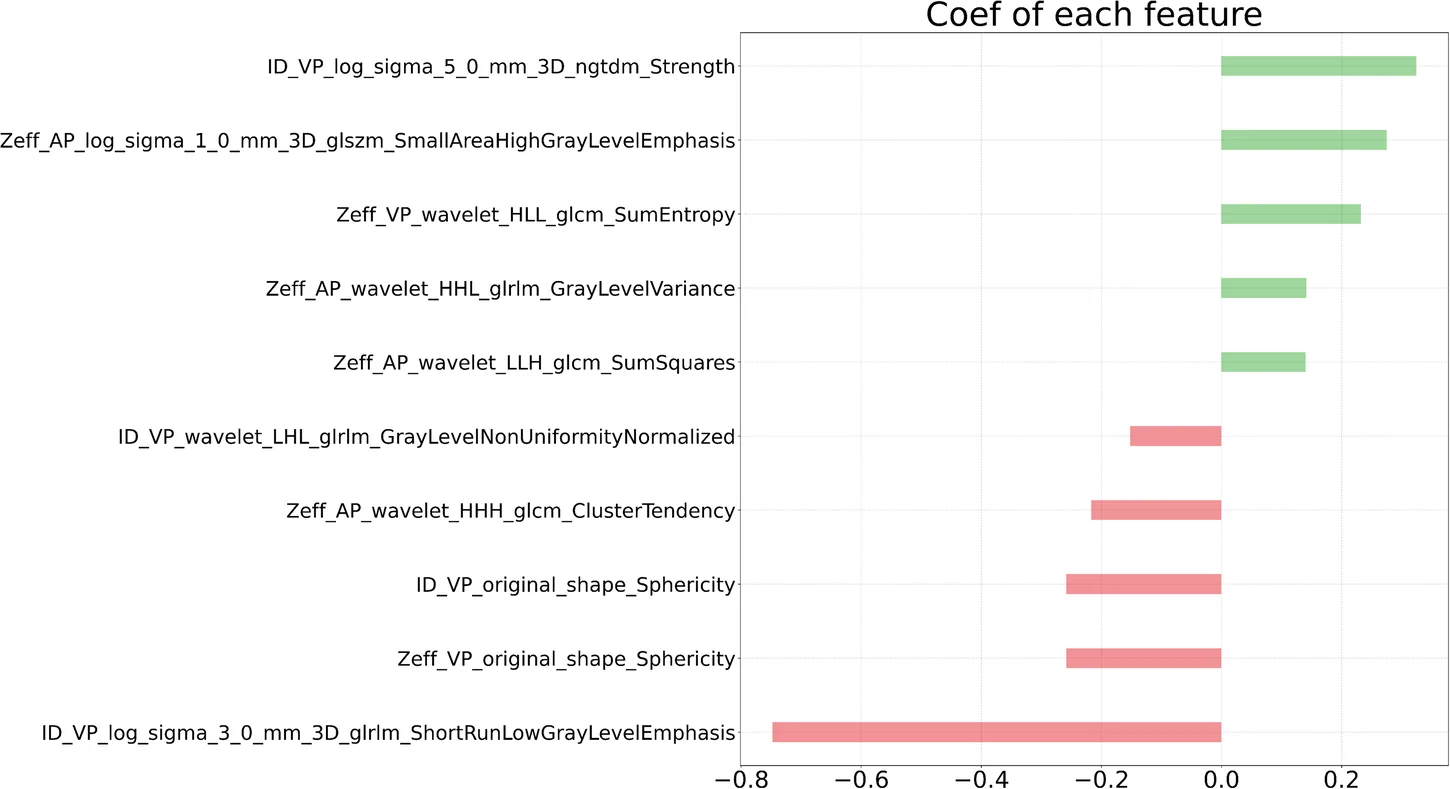
Feature coefficients of the radiomics model. AP, arterial phase; VP, venous phase; ID, iodine density; Zeff, effective atomic number

Based on repeated cross-validations, three optimal single-spectral models were selected: Zeff_AP, Zeff_VP and ID_VP. These models were subsequently compared with radiomics models from Conventional_VP and Conventional_AP. The diagnostic performance of the five single-spectral radiomics models is summarized in Table S2. In the training cohort, the single-spectral models based on Zeff_AP, Zeff_VP, and ID_VP all demonstrated superior diagnostic performance to conventional CT images. Among these, ID_VP achieved the highest AUC of 0.816 (95% CI: 0.755–0.876). Ten radiomic features derived from the top three performing models were integrated to construct a multiparametric radiomics model, yielding AUCs of 0.835 (95% CI: 0.776–0.893), 0.804 (95% CI: 0.694–0.913), and 0.772 (95% CI: 0.633–0.912) in the training, internal test, and external test cohorts, respectively. Detailed results are provided in Table 3. Feature coefficients and contribution weights are detailed in Fig. 3.

### Combined model

**Fig. 4:**
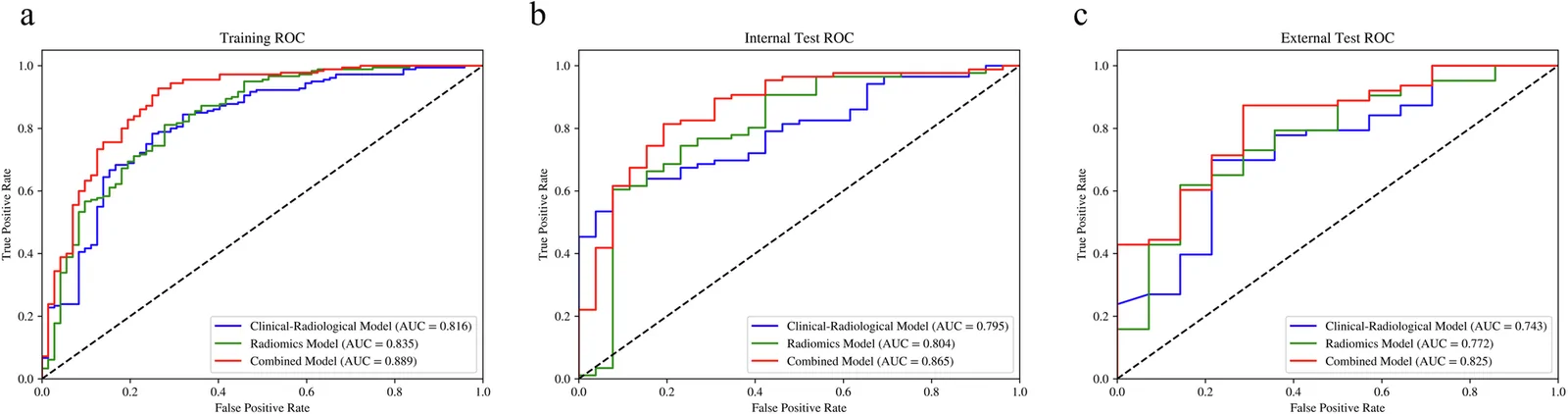
**a**–**c** ROC curves comparison for the clinical-radiological model, radiomics model, and combined model in the training, internal test, and external test sets. SPNs, solid pulmonary nodules; AUC, area under the curve; ROC, receiver operating characteristic

**Fig. 5:**
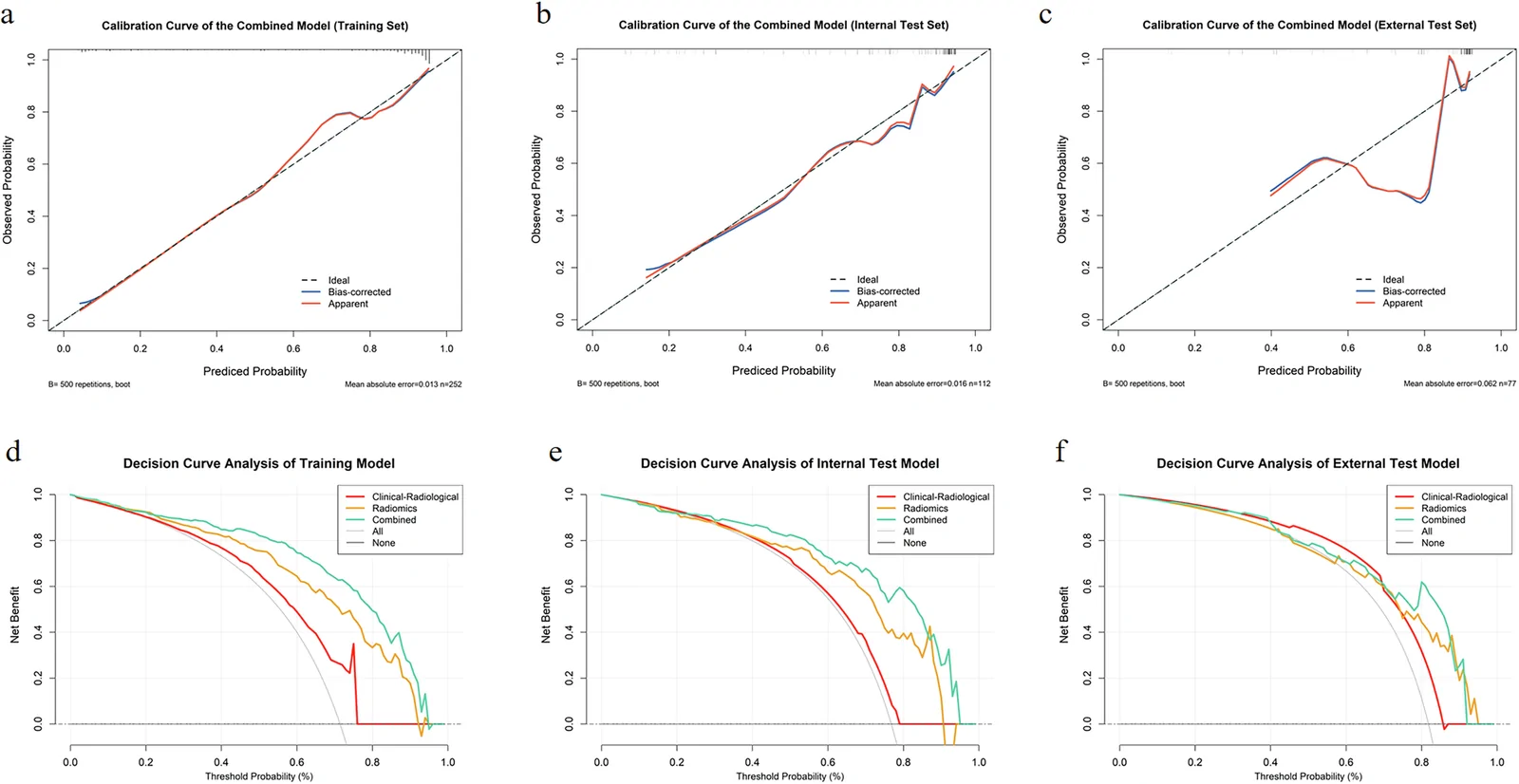
**a**–**c** Calibration curves for the combined model in the training, internal test, and external test sets. **d**–**f** Decision curves analysis for the clinical-radiological, radiomics model, and combined model in the training, internal test, and external test sets

The combined model, developed via logistic regression, incorporated two clinical-radiological variables (age and lobulation) alongside the multiparametric Radscore, and was illustrated as a nomogram (Fig. S1). Compared to the clinical-radiological model, the combined model achieved significantly higher diagnostic performance across all cohorts: training set (AUC: 0.889 vs. 0.816), internal test set (AUC: 0.865 vs. 0.795), and external test set (AUC: 0.825 vs. 0.743) (Fig. 4a–c and Table 3). The DeLong test comparing the combined model with the clinical-radiological model yielded a *p*-value of < 0.05 (Table S3). The Hosmer-Lemeshow tests confirmed good calibration across all datasets (*p* > 0.05; Fig. 5a–c), and DCA indicated that the model offered greater clinical benefit than alternative models (Fig. 5d–f).

### Subgroup analysis and radiologist comparison

The combined model maintained robust performance across sub-centimeter (≤ 10 mm) and larger (> 10 mm) nodules in both internal and external test cohorts. In the internal test cohort, the combined model achieved AUCs of 0.875 (95% CI: 0.683–1.000) for nodules ≤ 10 mm and 0.888 (95% CI: 0.802–0.975) for nodules > 10 mm. In the external test cohort, with AUCs of 1.000 and 0.794 (95% CI: 0.664, 0.923) for differentiating ≤ 10 mm and > 10 mm nodules, respectively (Table 4). When compared with two radiologists’ assessments in the internal test cohort, the combined model achieved higher specificity (0.808 vs. 0.692–0.731) and AUC (0.865 vs. 0.835–0.854), but lower sensitivity (0.802 vs. 0.977) (Table S4).

**Table 4:** Subgroup analysis of the combined model stratified by nodule diameter in the internal and external test sets

Model	AUC (95%CI)	Accuracy	Sensitivity	Specificity
Internal test set				
≤10 mm (*n* = 14)	0.875 (0.683 –1.000)	0.857	0.800	1.000
> 10 mm (*n* = 98)	0.888 (0.802–0.975)	0.806	0.803	0.818
External test set				
≤ 10 mm (*n* = 7)	1.000	1.000	1.000	1.000
> 10 mm (*n* = 70)	0.794 (0.664–0.923)	0.729	0.729	0.727

*AUC* area under the curve, *CI* confidence interval

## Discussion

This study developed and validated a machine learning model combining clinical-radiological features and DLCT radiomics to differentiate benign from malignant SPNs. The nomogram developed from this model leverages multiparameter data to enhance tissue differentiation and holds promise as a valuable preoperative noninvasive decision-support tool for the clinical evaluation of SPNs, particularly for indeterminate SPNs on conventional imaging.

In the realm of radiological diagnostics, clinical and conventional CT morphological characteristics play a pivotal role in distinguishing malignant from benign SPNs. Consistent with prior studies [25–27], our multivariate analysis identified age, diameter, lobulation, pleural indentation, and calcification as independent predictors of malignant nodules. Of these, age and lobulation were integrated into the final nomogram, with lobulation indicating spatial heterogeneity in tumor growth rates [27]. However, the interpretation of these conventional CT features remains inherently subjective and empirical, leading to significant inter-observer variability and suboptimal diagnostic accuracy, especially for indeterminate or small nodules [3, 28]. In contrast, radiomics enables objective quantification of tumor heterogeneity beyond visual assessment.

Conventional CT-based radiomics has been widely investigated for the differential diagnosis of SPNs, with reported AUCs ranging from 0.80 to 0.90 [8, 22, 29, 30]. However, these models rely primarily on morphological features or single-phase enhancement patterns, which may not capture hemodynamic characteristics or compositional heterogeneity. In contrast, DLCT can provide multiparametric spectral information, such as ID, Zeff, and VMI, enabling more comprehensive assessment of lesion perfusion and tissue composition. Thus, DLCT may offer additional diagnostic value for SPNs that remain indeterminate on conventional CT. To date, only a few studies have explored DLCT-based radiomics for distinguishing benign from malignant pulmonary nodules. Xu et al [22] developed a radiomics model based on 65 keV VMI, achieving an AUC > 0.90, while Liang et al [23] reported a 70 keV VMI model with an AUC of 0.877. Although these single-parameter studies provide preliminary evidence supporting the feasibility of DLCT radiomics, they are limited by relatively small sample sizes, insufficient numbers of benign cases, and a lack of external validation.

To address these limitations, the present study systematically evaluated 12 DLCT-derived parametric maps and developed a multiparametric DLCT radiomics model, which demonstrated robust diagnostic performance, with AUCs of 0.804 and 0.772 in the internal and external test sets, respectively. Building on this model, a combined model integrating clinical and radiological features was constructed, achieving AUCs of 0.865 and 0.825 on the internal and external test sets. This combined model demonstrates potential as a preoperative, noninvasive decision-support tool. Notably, our study was powered by a relatively large cohort (*n* = 441) and included both internal and external validation, which may enhance the generalizability of the findings compared with previous single-center studies. Furthermore, we specifically evaluated model performance in sub-centimeter nodules (≤ 10 mm), a subgroup underexplored in prior DLCT radiomics studies.

To differentiate benign from malignant SPNs, we selected ten discriminative features derived from ID_VP, Zeff_AP, and Zeff_VP to construct the radiomics model. These selected features included one morphological and eight textural parameters. Among them, log-sigma-3-0-mm-3D_glrlm_ShortRun LowGray Level Emphasis of ID_VP is the feature with the highest weight, with the established superiority of texture over morphology in capturing tumor complexity [3, 5]. ID_VP emerged as the optimal predictor, outperforming Zeff_AP and Zeff_VP [15]. This finding [15, 31] corroborates evidence that iodine concentration in VP correlates better with microvessel density, as contrast agents fully permeate the tumor interstitium during the VP, overcoming arterial-phase limitations caused by tortuous microvessels and incomplete basement membranes. While Zeff visualizes tissue composition, its susceptibility to iodine interference likely explains its suboptimal performance [15, 16]. The single-spectral DLCT radiomics model (ID_VP, Zeff_AP, and Zeff_VP) demonstrated superior diagnostic performance compared with traditional CT, consistent with previous studies [22, 31]. Furthermore, the radiomics model incorporating multi-parameter DLCT data showed further enhanced performance over the radiomics model based on single-spectral DLCT. This might be explained by the synergistic enhancement of diagnostic accuracy from the multi-parameter integration of ID and Zeff, which combined hemodynamic and structural heterogeneity information.

To evaluate clinical utility, the diagnostic accuracy of the combined model was compared with that of radiologists’ interpretations. The model showed slightly superior diagnostic performance (AUC = 0.865 vs. 0.835–0.854), higher specificity (0.808 vs. 0.692–0.731), but lower sensitivity (0.802 vs. 0.977). This discrepancy may reflect a clinical bias toward overcalling indeterminate nodules, which could result in unwarranted invasive interventions. Consequently, our model may serve as a reliable preoperative tool to help reduce unnecessary invasive interventions, with the potential to support clinical decision-making, particularly for indeterminate SPNs. Furthermore, this study demonstrates the combined model’s stable diagnostic performance across nodule-size subgroups, particularly for subcentimeter solid nodules (≤ 10 mm)—a diagnostically challenging cohort with subtle imaging features. The model demonstrated robust generalizability in both ≤ 10 mm and > 10 mm nodules, with AUCs of 0.875 and 0.888 (internal test), and 1.000 and 0.794 (external test), respectively. In the external test cohort, the AUC for nodules ≤ 10 mm reached 1.000, likely due to the small sample size (*n* = 9), highlighting the need for further validation.

However, this study has several limitations. First, despite a relatively large sample size (*n* = 441), its retrospective design introduces potential selection bias and may restrict the generalizability of the results. Second, the prevalence of malignancy in our cohort (71%) is notably higher than that in routine clinical practice. This reflects a high-risk population enriched for nodules with suspicious imaging or clinical features that underwent pathological verification. Consequently, this imbalance may introduce spectrum or verification bias and may limit its applicability to lower-risk populations. Therefore, the proposed model is suited for preoperative risk stratification in patients with indeterminate SPNs; prospective validation in screening or surveillance populations is warranted. Third, multiparametric DLCT may also introduce feature redundancy and increase model complexity due to correlations among different parametric maps. This may increase uncertainty in feature selection and the risk of overfitting, potentially affecting model generalizability and interpretability. Future studies should focus on robust feature selection strategies, standardized acquisition protocols, and multi-center external validation to assess model stability across different platforms and clinical settings.

## Conclusion

This study established and validated a multiparametric DLCT radiomics model combined with clinical and radiological features to distinguish preoperatively benign from malignant SPNs. It maintained robust performance across nodule sizes, including subcentimeter lesions, highlighting its potential as a noninvasive, reproducible tool to support preoperative risk stratification and reduce unnecessary interventions in the management of SPNs.

## Supplementary information

Supplementary information

## Data Availability

The data presented in this study are available on request from the corresponding author. The data are not publicly available due to hospital regulations.

## Abbreviations

AP: Arterial phase
AUC: Area under the curve
CI: Confidence interval
DCA: Decision curve analysis
DLCT: Dual-layer spectral CT
ED: Electron density
ICCs: Intraclass correlation coefficients
ID: Iodine density
mRMR: Minimum redundancy maximum relevance
OR: Odds ratio
ROC: Receiver operating characteristic
ROI: Regions of interest
SPNs: Solid pulmonary nodules
VMI: Virtual monoenergetic images
VP: Venous phase
Zeff: Effective atomic number

## References

[1] Bray F, Laversanne M, Sung H et al (2024) Global cancer statistics 2022: GLOBOCAN estimates of incidence and mortality worldwide for 36 cancers in 185 countries. CA Cancer J Clin 74:229–263 38572751 10.3322/caac.21834

[2] Rami-Porta R, Nishimura KK, Giroux DJ et al (2024) The International Association for the Study of Lung Cancer Lung Cancer Staging Project: proposals for revision of the TNM stage groups in the forthcoming (Ninth) edition of the TNM classification for lung cancer. J Thorac Oncol 19:1007–1027 38447919 10.1016/j.jtho.2024.02.011

[3] Liu J, Qi L, Wang Y et al (2024) Development of a combined radiomics and CT feature-based model for differentiating malignant from benign subcentimeter solid pulmonary nodules. Eur Radiol Exp 8:8 38228868 10.1186/s41747-023-00400-6 PMC10792135

[4] Feng B, Chen X, Chen Y et al (2020) Radiomics nomogram for preoperative differentiation of lung tuberculoma from adenocarcinoma in solitary pulmonary solid nodule. Eur J Radiol 128:109022 32371184 10.1016/j.ejrad.2020.109022

[5] Yankelevitz DF, Yip R, Henschke CI (2023) Impact of duration of diagnostic workup on prognosis for early lung cancer. J Thorac Oncol 18:527–537 36642158 10.1016/j.jtho.2022.12.020

[6] Liu J, Qi L, Wang Y et al (2023) Diagnostic performance of a deep learning-based method in differentiating malignant from benign subcentimeter (≤10 mm) solid pulmonary nodules. J Thorac Dis 15:5475–5484 37969262 10.21037/jtd-23-985 PMC10636433

[7] Zhong Y, Xu H, Zhang W et al (2022) Radiomic analysis of pulmonary nodules for distinguishing malignancy from benignancy: the value of using iodine maps from dual-energy computed tomography. J Comput Assist Tomogr 46:878–883 35830384 10.1097/RCT.0000000000001360

[8] Liu Z, Yang L, Liang J et al (2025) Radiomic features add incremental benefit to conventional radiological feature-based differential diagnosis of lung nodules. Eur Radiol 35:2968–2978 39604651 10.1007/s00330-024-11221-5

[9] El Alam R, Byrne SC, Hammer MM (2023) Rate of benign nodule resection in a lung cancer screening program. Clin Imaging 104:109984 37832324 10.1016/j.clinimag.2023.109984 PMC10783430

[10] Yu Y, Cheng JJ, Li JY et al (2020) Determining the invasiveness of pure ground-glass nodules using dual-energy spectral computed tomography. Transl Lung Cancer Res 9:484–495 32676312 10.21037/tlcr.2020.03.33 PMC7354160

[11] Lin Z, Liu Y, Xia C et al (2025) Dual-energy CT radiomics combined with quantitative parameters for differentiating lung adenocarcinoma from squamous cell carcinoma: a dual-center study. Acad Radiol 32:1675–1684 39327138 10.1016/j.acra.2024.09.024

[12] Ran J, Cao R, Cai J et al (2021) Development and validation of a nomogram for preoperative prediction of lymph node metastasis in lung adenocarcinoma based on radiomics signature and deep learning signature. Front Oncol 11:585942 33968715 10.3389/fonc.2021.585942 PMC8101496

[13] Sudarski S, Hagelstein C, Weis M et al (2015) Dual-energy snap-shot perfusion CT in suspect pulmonary nodules and masses and for lung cancer staging. Eur J Radiol 84:2393–2400 26454742 10.1016/j.ejrad.2015.09.024

[14] Choe J, Lee SM, Do KH et al (2019) Prognostic value of radiomic analysis of iodine overlay maps from dual-energy computed tomography in patients with resectable lung cancer. Eur Radiol 29:915–923 30054795 10.1007/s00330-018-5639-0

[15] He C, Liu J, Li Y et al (2022) Quantitative parameters of enhanced dual-energy computed tomography for differentiating lung cancers from benign lesions in solid pulmonary nodules. Front Oncol 12:1027985 36276069 10.3389/fonc.2022.1027985 PMC9582258

[16] Liu K, Wang M, Xu Y et al (2023) Value of spectral computed tomography-derived quantitative parameters based on full volume analysis in the diagnosis of benign/malignant and pathological subtypes of solitary pulmonary nodules. Quant Imaging Med Surg 13:3827–3840 37284111 10.21037/qims-22-979 PMC10240008

[17] Zhang G, Li S, Yang K et al (2022) The value of dual-energy spectral CT in differentiating solitary pulmonary tuberculosis and solitary lung adenocarcinoma. Front Oncol 12:1000028 36531032 10.3389/fonc.2022.1000028 PMC9748684

[18] Lambin P, Rios-Velazquez E, Leijenaar R et al (2012) Radiomics: extracting more information from medical images using advanced feature analysis. Eur J Cancer 48:441–446 22257792 10.1016/j.ejca.2011.11.036 PMC4533986

[19] Gillies RJ, Kinahan PE, Hricak H (2016) Radiomics: images are more than pictures, they are data. Radiology 278:563–577 26579733 10.1148/radiol.2015151169 PMC4734157

[20] Ni XQ, Yin HK, Fan GH, Shi D, Xu L, Jin D (2021) Differentiation of pulmonary sclerosing pneumocytoma from solid malignant pulmonary nodules by radiomic analysis on multiphasic CT. J Appl Clin Med Phys 22:158–164 33369106 10.1002/acm2.13154 PMC7882110

[21] Mao L, Chen H, Liang M et al (2019) Quantitative radiomic model for predicting malignancy of small solid pulmonary nodules detected by low-dose CT screening. Quant Imaging Med Surg 9:263–272 30976550 10.21037/qims.2019.02.02 PMC6414768

[22] Xu H, Zhu N, Yue Y et al (2023) Spectral CT-based radiomics signature for distinguishing malignant pulmonary nodules from benign. BMC Cancer 23:91 36703132 10.1186/s12885-023-10572-4 PMC9878920

[23] Liang G, Yu W, Liu SQ et al (2022) The value of radiomics based on dual-energy CT for differentiating benign from malignant solitary pulmonary nodules. BMC Med Imaging 22:95 35597900 10.1186/s12880-022-00824-3 PMC9123722

[24] Koo TK, Li MY (2016) A guideline of selecting and reporting intraclass correlation coefficients for reliability research. J Chiropr Med 15:155–163 27330520 10.1016/j.jcm.2016.02.012 PMC4913118

[25] Qu BQ, Wang Y, Pan YP, Cao PW, Deng XY (2024) The scoring system combined with radiomics and imaging features in predicting the malignant potential of incidental indeterminate small (< 20 mm) solid pulmonary nodules. BMC Med Imaging 24:234 39243018 10.1186/s12880-024-01413-2 PMC11380408

[26] He XQ, Huang XT, Luo TY, Liu X, Li Q (2024) The differential computed tomography features between small benign and malignant solid solitary pulmonary nodules with different sizes. Quant Imaging Med Surg 14:1348–1358 38415140 10.21037/qims-23-995 PMC10895103

[27] Xu DM, van Klaveren RJ, de Bock GH et al (2008) Limited value of shape, margin and CT density in the discrimination between benign and malignant screen detected solid pulmonary nodules of the NELSON trial. Eur J Radiol 68:347–352 17920800 10.1016/j.ejrad.2007.08.027

[28] Wen Y, Wu W, Liufu Y et al (2024) Differentiation of granulomatous nodules with lobulation and spiculation signs from solid lung adenocarcinomas using a CT deep learning model. BMC Cancer 24:875 39039511 10.1186/s12885-024-12611-0 PMC11265160

[29] Chen X, Feng B, Chen Y et al (2020) A CT-based radiomics nomogram for prediction of lung adenocarcinomas and granulomatous lesions in patient with solitary sub-centimeter solid nodules. Cancer Imaging 20:45 32641166 10.1186/s40644-020-00320-3 PMC7346427

[30] Li Q, Li X, Li XY, Huo JW, Lv FJ, Luo TY (2020) Spectral CT in lung cancer: usefulness of iodine concentration for evaluation of tumor angiogenesis and prognosis. AJR Am J Roentgenol 215:595–602 32569515 10.2214/AJR.19.22688

[31] Zheng XX, Ma YQ, Cui YQ et al (2024) Multiparameter spectral CT-based radiomics in predicting the expression of programmed death ligand 1 in non-small-cell lung cancer. Clin Radiol 79:e511–e523 38307814 10.1016/j.crad.2024.01.006

