# Supplementary material for: Noninvasive differentiation of benign and malignant solid pulmonary nodules using multiparameter dual-layer spectral CT radiomics
Source: Insights Imaging. 2026 Sep 4;17:219. doi: 10.1186/s13244-026-02380-8 (PMC13545195; doi:10.1186/s13244-026-02380-8)
Supplement: Supplementary file 1 [file 13244_2026_2380_MOESM1_ESM.pdf]

Non-invasive differentiation of benign and malignant solid pulmonary nodules using multiparameter dual-layer spectral CT radiomics

ELECTRONIC SUPPLEMENTARY MATERIAL

Noninvasive differentiation of benign and malignant solid pulmonary nodules using multiparameter dual-layer spectral CT radiomics

Appendix S1

CT protocol in Center 1: The scanning parameters were as follows: (I) 120 kVp tube voltage; (II) automatic mAs for tube current (140-250mAs); (III) pitch of 0.671 (IV) rotation time of 0.33s; (V) 64mm × 0.625 mm detector collimation. For contrast-enhanced CT, all patients received an intravenous injection of contrast agent Iopamidol (370 mg/mL) at a dose of 1.35 mL/kg and a flow rate of 3.0 mL/s via a power injector, followed by a 30 mL saline flush administered at the same rate. The arterial phase (AP) scan was triggered using bolus-tracking technology, with the venous phase (VP) scan commencing 40 seconds after the AP.

CT protocol in Center 2：The scanning parameters were as follows: (I) 120 kVp tube voltage; (II) automatic mAs for tube current (140-250mAs); (III) pitch of 0.671 (IV) rotation time of 0.33s; (V) 64mm × 0.625 mm detector collimation. The intravenous contrast agent Iomeron 400 (400 mg/mL; Bracco Imaging) was injected with a power injector at 1.2–1.3 mL/kg with a flow rate of 1.6–2.5 mL/s, followed by a 30-mL saline flush at the same flow rate. Arterial and venous phase scans were carried out at 35 s and 65 s after injection of the contrast agent, respectively.

Table S1 The histology of all enrolled benign and malignant lung nodules in the internal and external data sets

Malignancy	Histology	Number of nodules	
		Center 1 (n=364)	Center 2 (n=77)
Malignant nodules	Squamous cell carcinoma	30	9
	Adenocarcinoma	196	51
	Adenosquamous carcinoma	1	-
	Carcinoid	4	-
	Small cell lung carcinoma	9	-
	Large cell carcinoma	1	-
	Poorly differentiated carcinoma	2	-
	MALT lymphoma	3	-
	Neuroendocrine carcinoma	2	1
	Metastatic carcinoma	18	2
Benign nodules	Nonspecific inflammation	33	6
	Granulomatous inflammation	22	3
	Pulmonary hamartoma	20	3
	Sclerosing pneumocytoma	8	-
	Fungal infection	2	2
	A typical adenomatous hyperplasia	2	-
	Leiomyoma	1	-
	Hemangioma	1	-
	Bronchogenic cyst	1	-
	Bronchiolar adenoma	1	-
	Epithelioid hemangioendothelioma	1	-
	Chondroma	1	-
	Fibrotic nodule	5	-

MALT mucosa-associated lymphoid tissue

Table S2 Performance of Conv_AP, Conv_VP, Zeff_AP, Zeff_VP, and ID_VP Models

**Model**	**AUC (95%CI)**	**Accuracy**	**Sensitivity**	**Specificity**
Training cohort				
Conv_AP	0.725 (0.653-0.797)	0.746	0.817	0.569
Conv_VP	0.700 (0.625-0.775)	0.714	0.772	0.569
Zeff_AP Model	0.751 (0.685-0.817)	0.679	0.639	0.778
Zeff_VP Model	0.743 (0.670-0.816)	0.754	0.800	0.639
ID_VP Model	0.816 (0.755-0.876)	0.798	0.828	0.722
Internal test cohort				
Convl_AP	0.653 (0.535-0.771)	0.723	0.826	0.385
Conv_VP	0.667 (0.530-0.804)	0.714	0.779	0.500
Zeff_AP Model	0.687 (0.569-0.806)	0.616	0.593	0.692
Zeff_VP Model	0.666 (0.525-0.806)	0.768	0.837	0.538
ID_VP Model	0.746 (0.625-0.867)	0.741	0.767	0.654
External test cohort				
Conv_AP	0.707(0.574-0.841)	0.688	0.746	0.429
Conv_VP	0.604 (0.525-0.784)	0.429	0.349	0.786
Zeff_AP Model	0.622 (0.424-0.801)	0.377	0.302	0.714
Zeff_VP Model	0.625 (0.430-0.819)	0.636	0.635	0.643
ID_VP Model	0.749 (0.597-0.902)	0.571	0.540	0.714

AP arterial phase, *Conv* conventional, *VP* venous phase, *Zeff* effective atomic number, *ID* iodine density, *AUC* area under the curve, *CI* confidence interval,

Table S3 The Delong test among in the training, internal and external test cohort models

Model	Clinical	Zeff_AP	Zeff_VP	ID_VP	Conv_VP	Conv_AP	Radiomics	Combined
Training								
Clinical	/	0.0271	0.0239	0.97	0.0006	0.0012	0.4739	0.0016
Zeff_AP	/	/	0.7806	0.03	0.078	0.3181	0.0001	<0.0001
Zeff_VP	/	/	/	0	0.1027	0.5003	0.0001	<0.0001
ID_VP	/	/	/	/	0.0002	0.0032	0.1611	0.0006
Conv_VP	/	/	/	/	/	0.3845	<0.0001	<0.0001
Conv_AP	/	/	/	/	/	/	0.0002	<0.0001
Radiomics	/	/	/	/	/	/	/	0.0013
Combined	/	/	/	/	/	/	/	/
Internal test								
Clinical	/	0.4974	0.3641	0.7873	0.3982	0.1422	0.2619	0.0124
Zeff_AP	/	/	0.658	0.3786	0.6862	0.5761	0.037	0.0015
Zeff_VP	/	/	/	0.1744	0.9713	0.8577	0.0074	0.0007
ID_VP	/	/	/	/	0.2111	0.2259	0.0166	0.0014
Conv_VP	/	/	/	/	/	0.836	0.0183	0.0018
Conv_AP	/	/	/	/	/	/	0.0356	0.0018
Radiomics	/	/	/	/	/	/	/	0.0206
Combined	/	/	/	/	/	/	/	/
External test								
Clinical	/	0.9751	0.9906	0.1412	0.8487	0.3308	0.0973	0.0021
Zeff_AP	/	/	0.9819	0.1614	0.8894	0.144	0.0089	0.0573
Zeff_VP	/	/	/	0.176	0.8687	0.4049	0.1444	0.0266
ID_VP	/	/	/	/	0.1533	0.5447	0.6532	0.3857
Conv_VP	/	/	/	/	/	0.3065	0.1325	0.0305
Conv_AP	/	/	/	/	/	/	0.146	0.1462
Radiomics	/	/	/	/	/	/	/	0.5586
Combined	/	/	/	/	/	/	/	/

AP arterial phase, *VP* venous phase, *Zeff* effective atomic number, *ID* iodine density, *Conv* conventional.

$P < 0.05$ with significant differences.

Table S4 Predictive performance of combined model and radiologists in the internal test set

Model	AUC (95%CI)	Accuracy	Sensitivity	Specificity
Combined Model	0.865 (0.782-0.945)	0.804	0.802	0.808
Radiologist B	0.854 (0.774 -0.913)	0.920	0.977	0.731
Radiologist C	0.835 (0.753-0.898)	0.911	0.977	0.692

AUC area under the curve, *CI* confidence interval,

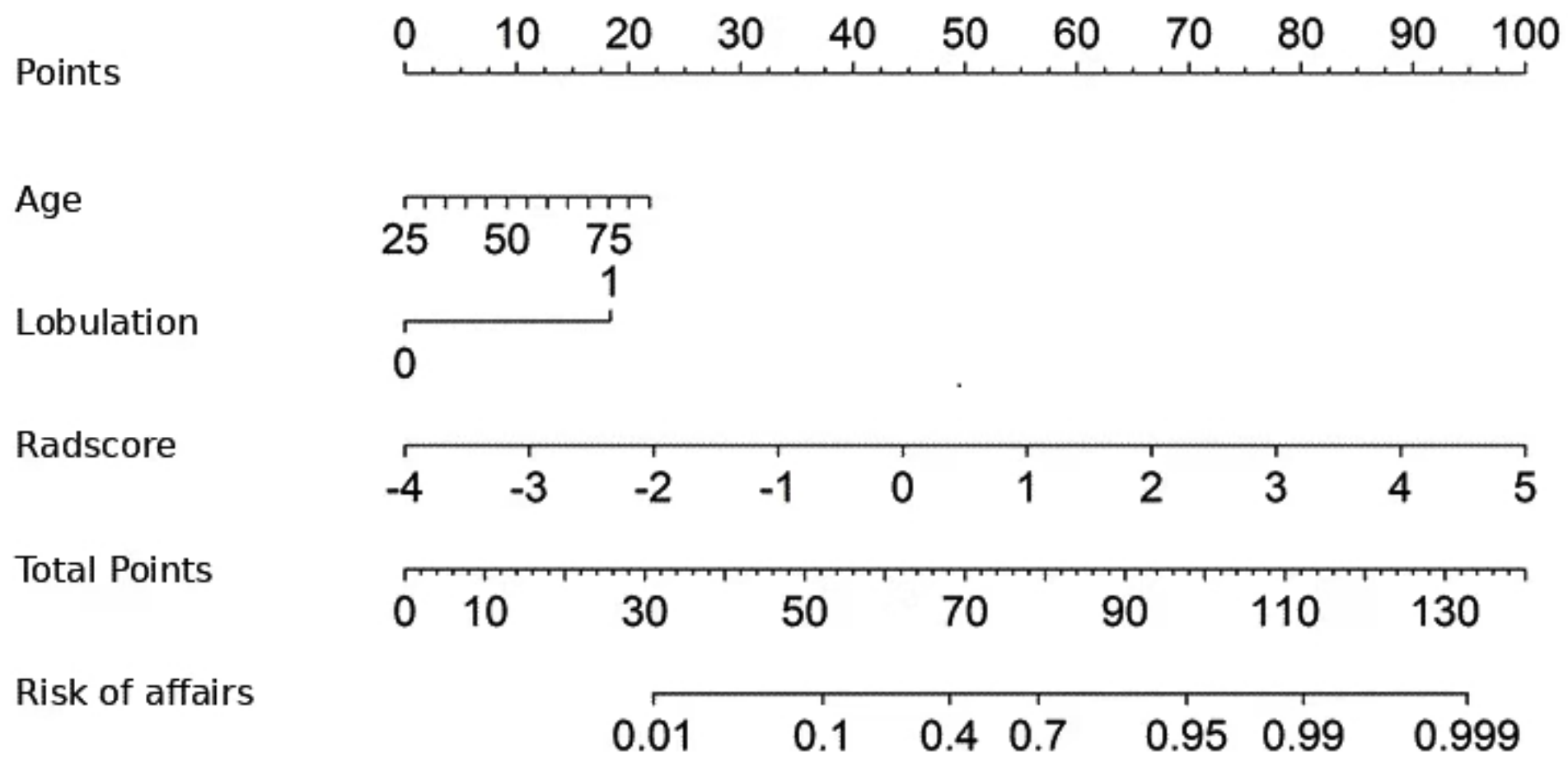

Figure S1. Nomogram for differentiating benign from malignant SPNs developed based on the training set.
